# “Omics” Technologies - What Have They Told Us About Uropathogenic *Escherichia coli* Fitness and Virulence During Urinary Tract Infection?

**DOI:** 10.3389/fcimb.2022.824039

**Published:** 2022-02-14

**Authors:** Sergi Torres-Puig, Vanesa García, Kristian Stærk, Thomas E. Andersen, Jakob Møller-Jensen, John E. Olsen, Ana Herrero-Fresno

**Affiliations:** ^1^ Department of Biochemistry and Molecular Biology, University of Southern Denmark, Odense, Denmark; ^2^ Department of Veterinary and Animal Sciences, Faculty of Health and Medical Sciences, University of Copenhagen, Frederiksberg, Denmark; ^3^ Laboratorio de Referencia de Escherichia coli (LREC), Departamento de Microbioloxía e Parasitoloxía, Facultade de Veterinaria, Universidade de Santiago de Compostela (USC), Lugo, Spain; ^4^ Research Unit of Clinical Microbiology, University of Southern Denmark and Odense University Hospital, Odense, Denmark

**Keywords:** UPEC, pathogenesis, fitness, virulence, UTIs, -omics

## Abstract

Uropathogenic *Escherichia coli* (UPEC) is the main etiological agent of urinary tract infection (UTI), a widespread infectious disease of great impact on human health. This is further emphasized by the rapidly increase in antimicrobial resistance in UPEC, which compromises UTI treatment. UPEC biology is highly complex since uropathogens must adopt extracellular and intracellular lifestyles and adapt to different niches in the host. In this context, the implementation of forefront ‘omics’ technologies has provided substantial insight into the understanding of UPEC pathogenesis, which has opened the doors for new therapeutics and prophylactics discovery programs. Thus, ‘omics’ technologies applied to studies of UPEC during UTI, or in models of UTI, have revealed extensive lists of factors that are important for the ability of UPEC to cause disease. The multitude of large ‘omics’ datasets that have been generated calls for scrutinized analysis of specific factors that may be of interest for further development of novel treatment strategies. In this review, we describe main UPEC determinants involved in UTI as estimated by ‘omics’ studies, and we compare prediction of factors across the different ‘omics’ technologies, with a focus on those that have been confirmed to be relevant under UTI-related conditions. We also discuss current challenges and future perspectives regarding analysis of data to provide an overview and better understanding of UPEC mechanisms involved in pathogenesis which should assist in the selection of target sites for future prophylaxis and treatment.

## 1 Introduction

### 1.1 Extraintestinal Pathogenic *Escherichia coli*

Extra intestinal pathogenic *Escherichia coli* (ExPEC) encompass those *E. coli* strains that are able to cause infection outside the intestine (Kaper et al., 2004). ExPEC isolates are facultative pathogens: they reside as commensals in the gut and belong to the normal microbiota, while outside the intestine are mainly related to neonatal meningitis and urinary tract infections (UTIs) in humans (Kohler and Dobrindt, 2011). They can be distinguished from the commensal and intestinal pathogenic *E. coli* by various genotyping methods, phylogrouping and multilocus sequence typing (MLST). Genotypically, they are characterized by harboring a broad range of virulence factors involved in cell adhesion, cell invasion, iron acquisition and evasion of the host immune response. Strains have to carry at least two of the following virulence genes to be classified as ExPEC: *papA* and/or *papC*, *sfa*/*foc*, *afa*/*draBC*, *kpsM II* and/or *iutA* (Peirano et al., 2013). In the past, the ExPEC category was divided into four pathotypes: uropathogenic *E. coli* (UPEC), neonatal meningitis associated *E. coli* (NMEC), sepsis associated *E. coli* (SEPEC), and avian pathogenic *E. coli* (APEC), based on the site of isolation and disease-association (Ewers et al., 2007). However, nowadays this classification is considered outdated, since isolates within the specific groups have been shown to cause infections at other sites (Dale and Woodford, 2015) and they overlap in the phylogenetic analysis (Jørgensen et al., 2019).

### 1.2 UPEC Pathogenesis During UTIs

A UTI is defined by the presence of significant amounts of bacteria in the urine accompanied by clinical symptoms of infection. UPEC is the primary cause of this disease accounting for up to 80% of all cases (Flores-Mireles et al., 2015). The classical model of UPEC-pathogenesis during UTI proposes that microorganisms of the intestinal microbiota colonize the periurethral space and ascend through the urethra into the bladder (Robino et al., 2014; Flores-Mireles et al., 2015; Terlizzi et al., 2017) Once in the bladder, the uropathogens may successfully colonize the bladder milieu, i.e. the urine (causing bacteriuria) and the uroepithelium. Here, bacteria undergo multiplication as planktonic cells as well as sessile, biofilm-embedded cells that eventually trigger bladder inflammation (cystitis). Clinical symptoms comprise increased urinary frequency, urge, dysuria, suprapubic pain, leukocyturia and hematuria (Flores-Mireles et al., 2015; Geerlings, 2016; Terlizzi et al., 2017). UPEC may subsequently multiply further and evade host immune responses and migrate from the bladder to the renal pelvis through the ureters causing pyelonephritis, that typically manifests with symptoms of both bladder and systemic inflammation (e.g., fever, chills, malaise). When reaching the kidneys, the uropathogens may gain access to the blood, leading to bacteriaemia (urosepsis). Bloodstream infections originating from the urinary tract is the leading cause of sepsis, and therefore pyelonephritis is a serious medical condition (Flores-Mireles et al., 2015; Terlizzi et al., 2017; Johnson and Russo, 2018).

In order to infect the bladder, UPEC expresses multiple virulence and fitness factors required for colonization, survival and growth in the urinary tract. Main virulence determinants include adhesins, flagella, iron-acquisition systems, and surface polysaccharide structures (Nielubowicz and Mobley, 2010; Bien et al., 2012; Subashchandrabose and Mobley, 2015a). Metabolic flexibility is also crucial for UPEC to succeed and cause disease (Mann et al., 2017). As demonstrated by several studies, UPEC is able to coordinate a complex orchestration of multiple factors both temporally and spatially in order to survive and persist in the adverse environment of the different niches within the urinary tract (Lane et al., 2007; Hannan et al., 2012; Sarkar et al., 2014; Conover et al., 2016b).

### 1.3 Epidemiology and Current UTI Management Strategies

In healthcare settings, UTIs represent the leading nosocomial infections accounting for 30-40% of all infections treated in hospitals (Stefaniuk et al., 2016). The main contributor to the high frequency of nosocomial UTIs is catheterization of the urinary bladder which dramatically increases the risk of UTI with an estimated 3% to 7% increase in probability of infection per day (Babich et al., 2017).

Outside hospitals, community-acquired UTIs globally affect more than 150 million people per year and represent an important health problem with high economic costs (Foxman et al., 2000). Although UTIs affect both genders, women are more likely to suffer from UTIs (with a female to male ratio of 50:1 in younger populations and 2:1 in old age) due to a shorter urethra closer to the rectal opening (Cove-Smith and Almond, 2007). It is estimated that over 50% of women will develop at least one episode of UTI during their lifetime, and one in three will have at least one symptomatic UTI requiring antimicrobial treatment by age 24 (Flores-Mireles et al., 2015; Mann et al., 2017). Recurrent urinary tract infections (rUTIs) represent a significant contributor to the high frequency of UTIs and become a problem in 25%-35% of women who experience an initial infection (Foxman et al., 2000; Foxman, 2014). The majority of these women are otherwise healthy with no urogenital abnormalities or other apparent risk factors and hence, the associated etiology of rUTIs remains undefined, thus hindering targeted treatment (Ejrnæs et al., 2011; Barber et al., 2013). Therefore, rUTIs represent a substantial social cost with a major impact on the quality of life (Glover et al., 2014).

Uncomplicated UTIs are often self-limiting but antibiotics rapidly and effectively relieve symptoms. Elderly with a UTI diagnosis who are not prescribed antibiotics have significantly higher risk of bacteremia and overall mortality (Gharbi et al., 2019). Despite the focus on antibiotic stewardship, antibiotic consumption has increased worldwide by 65% (measured in defined daily doses) from 2000 to 2015 (Klein et al., 2018). In a Danish study of over 2.3M systemic antibiotic prescriptions, UTI was the leading targeted disease accounting for 21% of prescriptions (Aabenhus et al., 2017). Prevention strategies of rUTIs are limited and are dominated by frequent- or long-term antibiotic prophylaxis that further increases antibiotic consumption (Glover et al., 2014). Also, 40-60% of women return to their previous infection pattern once prophylactic treatment has ceased (Stapleton and Stamm, 1997).

The large consumption of antibiotics in UTI treatment, even for short periods, drives the evolution of antimicrobial resistance (AMR) among uropathogens including UPEC (Ronald, 2003; Carlet, 2012; Ventola, 2015). Recently, there has been an alarming increase in AMR among UPEC strains towards more than 70% of the relevant drugs, including those defined by WHO as highly critical for human health (Terlizzi et al., 2017; Mazzariol et al., 2017). Due to economic and regulatory obstacles, the development of new antibiotics has stalled, leading to an antibiotic crisis where infections such as UTIs are becoming an increasing threat to human health (Spellberg, 2014; Ventola, 2015).

Without novel antibiotics in the pipeline and a growing concern regarding AMR in rUTIs, many non-antibiotics modalities to prevent and treat UTIs are being explored with the main focus on UPEC (Wawrysiuk et al., 2019). Putative alternative measures for UTI treatment include behavioral changes, dietary supplementation (such as Chinese herbal medicines and cranberry products in the form of juices or tablets), nonsteroidal anti-inflammatory drugs, probiotics (oral and vaginal), D-mannose, methenamine hippurate, estrogens, intravesical lycosaminoglycans, immunostimulants, vaccines and bacterial interference (i.e. prophylactic bladder-colonization) with non-virulent bacteria (Sihra et al., 2018). In several novel interventions, specific virulence genes of UPEC are being targeted to interrupt central pathogenic steps during the natural course of a UTI. For example, proanthocyanidins (PAC), the active ingredients of cranberry juice, have long been explored to fight UTIs. By blocking P fimbriae, an important adhesins of UPEC, PAC inhibit bacterial adhesion to bladder epithelial cells *in vitro* (Howell et al., 2005; Gupta et al., 2007; Howell, 2007). Although recommended for rUTI prevention by the American Urological Association, the clinical impact of cranberry-derived products on UTI remains controversial as concluded in a recent Cochrane study (Jepson et al., 2012; Anger et al., 2019).

Type-1 fimbriae are crucial UPEC virulence factors and the main appendage responsible for UPEC adhesion to bladder epithelial cells. As such, these fimbriae have been investigated as target for non-antibiotic treatments (Stærk et al., 2016; Kyriakides et al., 2020; Scribano et al., 2020). FimH, the Type-1 fimbria tip adhesin, binds to highly mannosylated uroplakin receptors of bladder epithelial cells, and D-mannose prevents UPEC adhesion through a competitive inhibition mechanism (Scribano et al., 2020). Unlike cranberry products, evidence mounts that D-mannose reduces the incidence of rUTI in humans (Kyriakides et al., 2020; Scribano et al., 2020). Chemically engineered high-affinity mannose analogues (mannosides) show similar results in murine models of UTI (Guiton et al., 2012). A combination of several of the mentioned agents might be the most effective way to reduce the rate of UTIs, thus avoiding the overuse of classic antimicrobials (Sihra et al., 2018).

Research into the underlying molecular mechanisms of bacterial adherence and invasion should allow for identification of novel prevention and treatment strategies. Moreover, increasing our knowledge on bacterial population dynamics in the urinary tract should increase our understanding of the mechanisms that lead to UTIs and rUTIs. Several studies indicate that bacteria, even of the same bacterial clone, may exist as subpopulations in different niches in the urinary tract, exhibiting highly diverse genetic expression, phenotypes and growth behavior (Stærk et al., 2016). Deeper characterization of these population dynamics may dramatically improve our ability to identify novel targets and design effective prophylactic strategies.

## 2 UPEC Under the Magnifying Glass: Use of ‘Omics’ Technologies to Uncover Virulence and Fitness Factors

High-throughput technologies, also known as ‘omics’, have deeply transformed the field of molecular microbiology by enabling a complete characterization of complex cellular responses (Franzosa et al., 2015; Gutleben et al., 2018). Comparative genomics and genome-wide transcriptional analyses as well as protein expression profiling have immensely benefitted from the advances in next generation sequencing (NGS) technologies and mass spectrometry, respectively, coupled with complex integrative bioinformatics analyses. However, despite all the advantages that these holistic high-throughput techniques represent for the field, more traditional approaches are still performed to validate the often-challenging amount of data that is obtained from ‘omics’ experiments.

Whole genome sequencing has become an essential tool for identifying virulence mechanisms and survival strategies of bacterial pathogens. Since the first bacterial whole-genome drafts were obtained 25 years ago (Fleischmann et al., 1995; Fraser et al., 1995), thousands of bacterial genomes have been sequenced including those from laboratory reference strains and clinical isolates, and they have been made available in public databases. This wealth of sequence information has made the identification of pathogen-specific genes possible through simple genomic comparison with non-pathogenic reference strains. In the case of UPEC, the first studies using sequenced genomes linked specific genomic regions to pathogenesis and virulence (Welch et al., 2002; Lloyd et al., 2007), as it is discussed later in this review. Further refinement of the sequencing technology has enabled the establishment of the UPEC methylome using Single Molecule Real Time (SMRT) sequencing (Forde et al., 2015) as well as massive parallel determination of transposon-insertion sites *via* transposon-insertion sequencing (TIS), a powerful technique used for querying bacterial genomes (Hadjifrangiskou et al., 2012; Phan et al., 2013; Barquist et al., 2013). As it will be discussed later, TIS represents one of the most efficient and widely used tools for studying functional genomics in UPEC in the last decade. However, as shown for most of the ‘omics’ approaches, the vast amount of generated (predicted) data needs further validation using traditional methods and may benefit from additional experiments to test individual mutants in order to rule out gene function compensation (transcomplementation) or polar effects caused by individual Tn-insertions (Cain et al., 2020). Despite that, the TIS protocol is undergoing continuous improvement, and the combination of TIS with other traditional techniques guarantees the beginning of a new golden age in the field of functional genomics. Overall, the increasing number of available fully sequenced genomes together with more detailed gene expression profiling should pave the way for UPEC genomics and clinical applications in the future (Bertelli and Greub, 2013). In addition, the development of new techniques combining NGS with chromatin immunoprecipitation, also known as ChIP, has permitted the functional investigation of DNA binding proteins. One significant example in UPEC was the determination of the NsrR regulon in CFT073 using this technique (Mehta et al., 2015).

In a similar manner, the development of RNA sequencing (RNAseq) has had a significant impact in the study of bacterial transcriptional responses. In the early ‘omics’ era, the majority of global gene expression analyses in UPEC were performed using dense strain-specific microarrays (Seed and Hultgren, 2005; Hagan et al., 2010). However, the dependence on microarray target probes to detect transcription was rapidly overcome by using the RNAseq technology, which also allowed the discovery of new non-coding transcripts with higher specificity (Croucher and Thomson, 2010). These advantages have led to the full replacement of microarrays by NGS during the past decade and facilitated novel holistic transcriptional studies that enabled the discovery of new virulence and fitness factors in UPEC (Bielecki et al., 2014; Subashchandrabose et al., 2014). Despite the sequencing power offered by RNAseq, the large datasets generated pose statistical challenges that can result in non-specific hits and high false-positive rates (Rajkumar et al., 2015). Therefore, it is often crucial to validate RNAseq data using other methods such as RT-qPCR (Nagalakshmi et al., 2008; Croucher and Thomson, 2010; Fang and Cui, 2011). In the recent years, modifications of the classic RNAseq protocols have been developed to allow the functional study of small RNAs (MAP- and GRIL-seq) or RNA-binding proteins (RIP- and RIL-seq) (Saliba et al., 2017). Most of these techniques have been initially developed in *E. coli* and are expected to generate new important insight into the molecular mechanisms underlying UPEC infection of the urinary tract.

Determining the proteome, i.e. the full protein complement of bacterial cells under specific conditions, has become a widely used approach for *de novo* identification of bacterial virulence factors and a complementary methodology for validation of genetic studies (Callister et al., 2008; Pérez-Llarena and Bou, 2016). From the early 2D gel electrophoresis to the more recent automated and quantitative mass spectrometry approaches, the field of proteomics is in constant evolution to the point where a future of proteomics beyond mass spectrometry and its inherent limitations is becoming realistic (Timp and Timp, 2020). In UPEC, proteomics has been mainly used to assess protein composition of outer membranes (Hagan and Mobley, 2007; Wurpel et al., 2015) as well as the identification of novel adherence factors (Wurpel et al., 2016).

Mass spectrometry has also been used in the development of other important ‘omics’ techniques, such as metabolomics, lipidomics, metallomics, and immunoproteomics to study metabolic networks, lipid identification, metal acquisition and homeostasis, as well as vaccine development. These emerging techniques have already generated valuable contributions to our understanding of UPEC pathogenesis such as: the determination of the interactive metabolome between UPEC and human urine (Su et al., 2016b), the identification of oxylipid mediators of inflammation (Packiriswamy et al., 2017), metal homeostasis (Koh et al., 2015; Subashchandrabose and Mobley, 2015b), or the identification of conserved outer membrane antigens that might represent vaccine targets against UPEC infection (Hagan and Mobley, 2007), to name a few.

**Figure 1:**
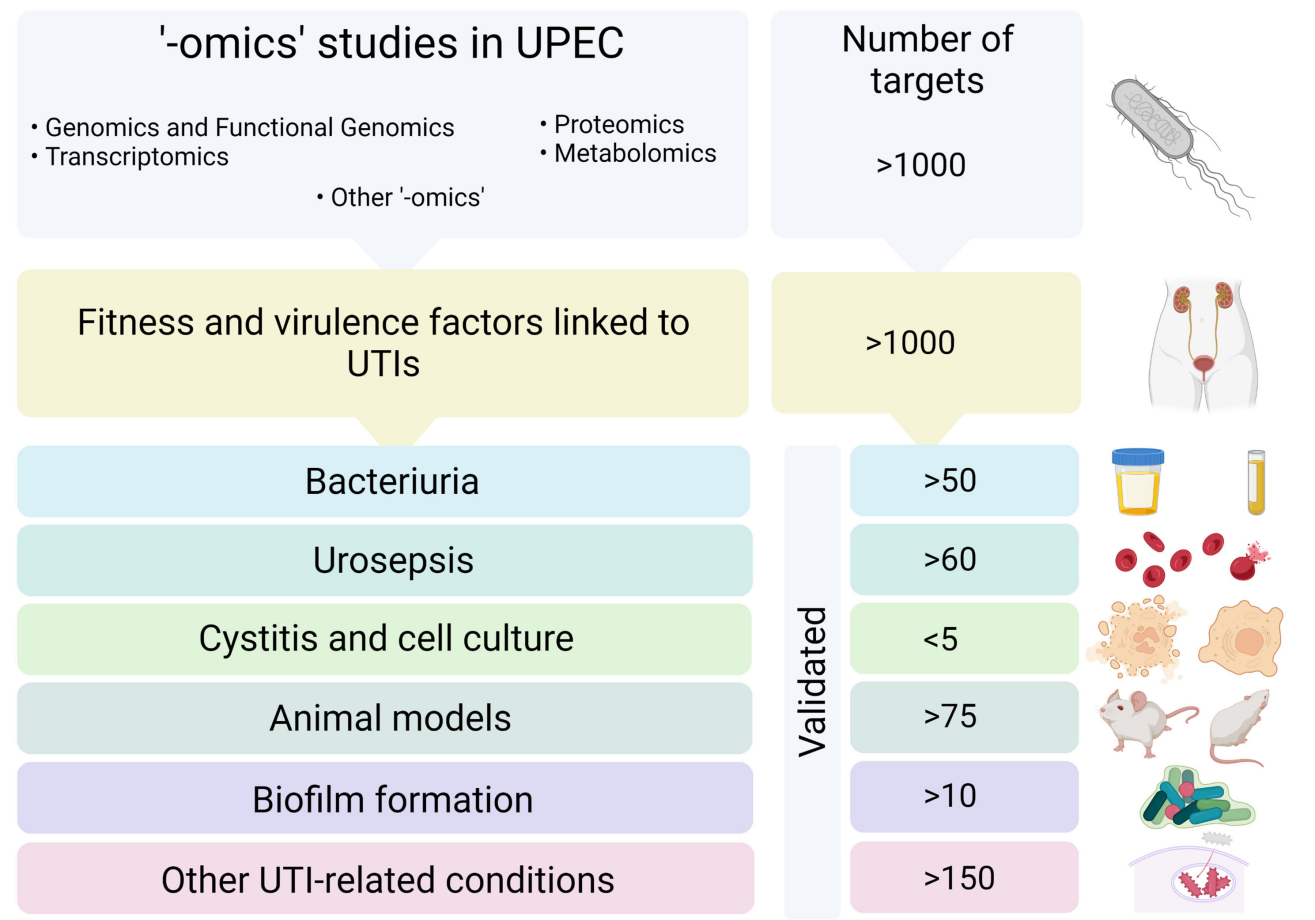
Scheme of the workflow followed in this review. Selected articles based on ‘-omics’ technologies applied to UPEC were were scrutinized in order to identify fitness and virulence factors involved in UTIs. The identified factors were classified according to the stage of a UTI where they were involved in and validated targets were cross-referenced and tabulated. Created with BioRender.com.

Implementation of ‘omics’ approaches has allowed a better understanding on how UPEC monitors the expression of the complex network of virulence and fitness factors and has provided new knowledge on the biological mechanisms that underlie UPEC pathogenesis. This is crucial in order to explore novel treatment and prevention strategies that will help overcome the problem of AMR development among UPEC. The large datasets generated call for a detailed analysis and scrutiny to understand which factors contribute the most to the development of UTIs, and not least to understand how the different factors interact between each other during the infection process. In the following, we review UPEC virulence and fitness determinants, which have been identified by the ‘omics’ approaches as important for development of UTIs with focus on those factors that have been confirmed by follow up studies (**Figure 1**). Furthermore, we discuss current challenges and future perspectives to better comprehend and analyze data revealed from ‘omics’ approaches.

## 3 Whole Genome Sequencing and Comparative Genomics of UPEC Strains

NGS has become the major revolution in bacterial genomics during the last decade. Despite being among the most well characterized microorganisms, pathogenic *E. coli* strains have many unique genomic traits that were poorly understood before the widespread application of ‘omics’ techniques. Whole genome sequencing of reference *E. coli* strains, both commensal and uropathogenic, and further comparative genomic analysis have revealed many putative virulence factors such as phase-switch mechanisms, toxins, a variable number of fimbrial adhesin families and iron acquisition systems, distributed in large pathogenicity islands (PAIs) in the UPEC genomes (Welch et al., 2002; Brzuszkiewicz et al., 2006; Chen et al., 2006; Klein and Gitai, 2013; Forde et al., 2014). The distinct codon usage and the function of genes located in these PAIs indicated that they have been acquired by the different UPEC strains *via* independent horizontal gene transfer events. Variation in these PAIs have been associated to virulence potential and persistence in chronic infections (Hochhut et al., 2006; Lloyd et al., 2007) and PAIs are believed to be responsible for the high adaptability of UPEC that enables colonization of different niches and provides with an excellent toolset to excel in ascending infections (Firoozeh et al., 2014).

The availability of fully sequenced UPEC genomes allowed the conduction of comparative genomics studies to identify key genes important for pathogenesis. Chen et al. used bioinformatics tools to find genes positively selected among seven different UPEC genomes and found that genes involved in cell wall/membrane biogenesis, secondary metabolites biosynthesis as well as replication, recombination and repair were common to all strains tested (Chen et al., 2006). The limitation of this first study was the short number of available sequences, which constrained the potential of the computational pipeline used. However, nowadays, the number of sequenced isolates has grown exponentially, which allows more ambitious comparative genomics studies between strains (Subashchandrabose et al., 2013a; Lo et al., 2015). Results from such studies will be of great value to better understand the virulence potential of UPEC and unravel unknown traits of the biology of *E. coli* as a causative agent of UTIs.

### 3.1 The Golden Age of Functional Genomics

In parallel to whole genome sequencing, bioinformatics analysis of fully sequenced genomes rapidly became a powerful tool to establish hypotheses on the link between the presence of genes and certain (virulent) phenotypes. However, despite the ability to study gene-transcription using RNAseq, it was not until the development of TIS that the field of functional genomics reached a major milestone (van Opijnen and Camilli, 2013). TIS combines traditional transposon mutagenesis with genome-wide targeted sequencing; it allows the analysis of hundreds of thousands of mutants at the same time by performing NGS of saturated transposon libraries in a forward genetics manner (Langridge et al., 2009). Moreover, the technique enables the rapid identification of essential and fitness genes under any assayable particular growth condition with outstanding resolution (Barquist et al., 2013). In addition, the implementation of accessible bioinformatics tools such as the Bio-Tradis pipeline (Barquist et al., 2016) to analyze the large amounts of data resulting from TIS studies, in this particular case from a specific TIS-based approach (TraDIS), has facilitated the interpretation and comparison of results from different laboratories (Barquist et al., 2016). However, as it will be discussed below, even this powerful tool is not without limitations, especially for studies modelling infections *in vivo*, where bottleneck effects can cause a significant distortion in the number of surviving clones (Walters et al., 2012).

#### 3.1.1 Identification of UPEC Factors Involved in Different Stages of a UTI

TIS has been widely used to gain knowledge on UPEC virulence and fitness factors and to discover novel therapeutic targets to fight UTIs by analyzing the bacterial ability to colonize different niches. An important factor contributing to UPEC pathogenesis is the ability to form biofilms. One of the first reported studies using TIS in UPEC aimed to determine important genes for biofilm formation in the UPEC strain UTI89 (Hadjifrangiskou et al., 2012). The mutant library obtained consisted of 44,386 transposon mutants (Tn-mutants), 6,144 of which were manually screened for defects in three different *in vitro* biofilm conditions: Luria broth (LB)-polyvinyl chloride (PVC), YESCA (yeast extract-Casamino Acids)-PVC, and YESCA-pellicle that are dependent on type 1 pili and curli. Flagella were found to be relevant in all three conditions. Some mutants tested showed biofilm defects in all three conditions but without significant effects on the expression of type 1 pili, curli, or flagella. In addition, results showed that some of the identified factors were also required for infection of the murine UTI model and formation of intracellular bacterial communities (IBC´s) (**Table 1**).

**Table 1:** Validated genes identified by TraDIS.

Gene	Function (COG)^5^	Validation study						UPEC strain	Reference
		Urine (bacteriuria)	Serum (urosepsis)	Infection of cells (cystitys)	Animal model(bladder, kidney)	Biofilm formation	Other		
*purH*	F					X		**UTI89**	(Hadjifrangiskou et al., 2012)^6^ ***cvpA*** (Shaffer et al., 2017) ***visC*** (Floyd et al., 2016)
*UTI89_C3813*	–					X*			
***cvpA***	M,F					**X**			
*plsX*	I					X			
*carB*	E,F					X			
***visC* (*ubiI*)**	C,H				Murine bladder	**X**			
*rfe*	M				Murine bladder	X			
*acrA^1^*	V,M		X*				LPS*/MI*	EC958 ***waaL*** (CFT073) ***rfaH*** (536)	(Phan et al., 2013) ***waaL*** (Sarkar et al., 2014) ***rfaH*** (Nagy et al., 2002)
*EC958_2373^1^*	R		X*				LPS*/MI*		
*dnaJ*	O		X				LPS*/MI*		
*acnB*	C		X				LPS*/MI*		
*EC958_0460*	–		X				LPS/MI*		
*EC958_0461*	–		X				LPS/MI*		
*nagC*	K,G		X				LPS*/MI*		
*nagA*	G		X				LPS/MI		
*pgm*	G		X				LPS/MI		
*tolQ*	U		X				LPS*/MI		
*tolA*	M		X				LPS*/MI		
*galE*	M		X				LPS/MI*		
*EC958_1112*	–		X				LPS/MI*		
*EC958_1114*	–		X				LPS*/MI*		
*lpp*	M		X				LPS*/MI		
*wzz*	M		X				LPS/MI*		
*EC958_2371*	G		X				LPS/MI		
*rmlC*	M		X				LPS/MI		
*rmlA*	M		X				LPS/MI*		
*rmlD*	M		X				LPS/MI*		
*rmlB*	M		X				LPS/MI*		
*gmm*	M		X				LPS*/MI*		
*wcaF*	R		X				LPS/MI		
*arnD*	M,G		X				LPS*/MI		
*arnE*	M,G		X				LPS*/MI*		
*arnF*	M,G		X				LPS*/MI*		
*dedA*	M		X				LPS*/MI*		
*bamB*	M		X				LPS*/MI		
*greA*	K		X				LPS*/MI		
*sspA*	D		X				LPS*/MI		
***waaL***	M		**X**				LPS/MI		
*waaK*	M		X				LPS/MI		
*waaY*	M		X				LPS/MI		
*waaJ*	M		X				LPS/MI		
*waaI*	M		X				LPS/MI		
*waaB*	M		X				LPS/MI*		
*waaP*	M		X				LPS/MI		
*waaG*	M		X				LPS/MI		
*pyrE*	F		X				LPS*/MI*		
*wecA*	M		X				LPS/MI		
*wzzE*	M		X				LPS/MI		
*wecD*	K		X				LPS/MI		
*wecE*	E		X				LPS*/MI*		
*wzyE*	S		X*				LPS*/MI		
*wecF*	C		X				LPS*/MI		
***rfaH***	K		**X**				**LPS**/MI		
*dsbA*	O		X				LPS*/MI		
*pgi*	G		X				LPS*/MI		
*miaA*	J		X*				LPS*/MI		
*fbp*	G		X				LPS*/MI*		
*nhaA*	C,P		X*				LPS*/MI*		
*tolB*	U		X*	X^7^	Bladder^7^/kidneys^7^		LPS*/MI*		
*wcaI*	G		X*				LPS*/MI*		
*truA*	J		X*				LPS*/MI*		
*nusA*	K		X*				LPS*/MI*		
*wzxE*	M		X*				LPS*/MI*		
*pgaABCD*	N				Bladder/spleen/kidneys			CFT073	(Subashchandrabose et al., 2013b)
*pilVS*	N,W				Bladder/spleen/liver/kidneys				
*sapACF*	V				Spleen/liver				
*oppA*	E				Spleen/liver				
*pic*	M				Spleen/liver				
*vat*	S				Spleen/liver				
*c1220*	E				Spleen/liver				
*yddB*	P				Spleen/liver				
*pqqL*	L				Spleen/liver				
*tppB*	E				Bladder/spleen*/liver*/kidneys				
*ycgF*	T				Bladder/spleen*/liver*/kidneys				
***lrhA***	K						Hyper-motility	EC958 **CFT073**	(Kakkanat et al., 2017) ***lrhA, ydiV, lrp*** (Simms and Mobley, 2008a) ***ydiV*** (Spurbeck et al., 2013)
*ihfA*	L						Hyper-motility		
***ydiV***	T						Hyper-motility		
***lrp***	K						Hyper-motility		
*mprA*	K						Hyper-motility		
*hemK*	J						Hyper-motility		
*yjeA*	J						Hyper-motility		
*EC958_1546^2,3^*	–						Hyper-motility		
*mprA*	K						Capsule synthesis	PA45B UTI89 CFT073	(Goh et al., 2017)
*lrhA* ^2^	K						Capsule synthesis		
*rfaH*	K						Capsule synthesis		
*typA*	T						Capsule synthesis		
*argP*	K						Capsule synthesis*		
*ygaZ*	E						Capsule synthesis*		
*fad_1*	?						Capsule synthesis*		
*wecA*	M						Capsule synthesis*		
*kbl_2*	???						Capsule synthesis*		
*wzyE*	S						Capsule synthesis*		
*glpT_2*	G,P						Capsule synthesis*		
*glcA*	C,P						Capsule synthesis*		
*PA45B_03292*	–						Capsule synthesis*		
*yghG*	M						Capsule synthesis*		
*rbfA*	J						Capsule synthesis*		
*alaA*	E						Capsule synthesis*		
*fadL*	I						Growth in mucus	UPEC F11	(Russell et al., 2017)
*fbp*	G						Growth in mucus		
*glpG*	S				Mouse gut		Growth in mucus		
*aapA*	–						Growth in mucus*		
*cof*	H,R						Hemolysis	CFT073	(Murthy et al., 2018)
*hlyA*	Q						Hemolysis		
*wbbL^4^*	M						Colicin resistance	EC958	(Sharp et al., 2019)
*hlyCABD*	O						Hemolysis (increased)	S65SC	(Nhu et al., 2019)
*rfaE*	H						Hemolysis		
*waaC*	M						Hemolysis		
*waaF*	M						Hemolysis		
*waaG*	M						Hemolysis		
*dnaJ*	O						Hemolysis		
*yqhG*	D				Human bladder cells, bladder and kidney (mice)		T1P expression, oxidative stress, hyper-motility	CFT073	(Bessaiah et al., 2019)
*pstB*	P						Zn resistance	EC958	(Stocks et al., 2019)
*cpxR*	K,T						Zn resistance		
*ompR*	K,T						Zn resistance		
*yciB*	D						Zn resistance		
*hflC*	O						Zn resistance		
*hflK*	O						Zn resistance		
*minC*	D						Zn resistance		
*tolB*	U						Zn resistance		
*zntA*	P						Zn resistance		
*ftsX*	D						Zn resistance		
*minD*	D						Zn resistance		
*envC*	D						Zn resistance		
*ldcA*	M						Zn resistance		
*yibN*	P						Zn resistance		
*fabF*	I						Zn resistance*		
*yajC*	U						Zn resistance*		
*envZ*	T						Zn resistance		
*zntR*	K						Zn resistance		
*ppk*	C						Zn resistance		
*yjjY*	S						Zn resistance*		
*rlmE*	J						Zn resistance*		
*ihfA*	L						Zn resistance*		
*nouK*	C						Zn resistance*		
*rpmG*	J						Zn resistance (tolerance)		
*apaG*	P						Zn resistance (tolerance)		
*arfB*	J						Zn resistance*		
*rfaH*	K						Zn resistance (tolerance)		
*ihfB*	L						Zn resistance*		
*apaH*	T						Zn resistance (tolerance)		
*pitA*	P						Zn resistance (tolerance)		
*ftsN^1^*	D						Cefotaxime susceptibility*	EC958	(Phan et al., 2020)
*slt*	M						Cefotaxime susceptibility		
*tatB*	U						Cefotaxime susceptibility		
*envC*	D						Cefotaxime susceptibility		
*bla_CMY-23_*	V						Cefotaxime susceptibility		
*ampG*	G,E,P,R						Cefotaxime susceptibility		
*dacA*	M						Cefotaxime susceptibility		
*yfiH*	P						Cefotaxime susceptibility		
*ftsP*	D						Cefotaxime susceptibility		
*tatC*	U						Cefotaxime susceptibility		
*uvrA*	L						Cefotaxime susceptibility		
*mrcB*	M						Cefotaxime susceptibility		
*damX*	D						Cefotaxime susceptibility		
*efp*	J	X			Bladder/kidneys			CFT073 **Ec83972**	(Shea et al., 2020) **(** Vejborg et al., 2012 **)**
*rfe*	M	X			Bladder/kidneys*				
***carB***	E,F	**X**			Bladder/kidneys*				
*flhB*	N	X			Bladder/kidneys*				
*yihE*	T	X			Bladder/kidneys*				
*yfgM*	T	X			Bladder/kidneys*				
*purB*	F	X			Bladder/kidneys				
*cusB*	V,M	X			Bladder*/kidneys*				
*mdoH*	M,G	X*			Bladder*/kidneys*				
*yjbB*	P	X*			Bladder*/kidneys*				
*pshM*	U	X*			Bladder*/kidneys*				
*yfjO*	S	X*			Bladder*/kidneys*				
*pncB*	H	Human*							
*yigP*	H	Human							
*gidA*	J	Human*							
*msbB*	I	Human*							
*thdF*	J	Human*							
*ubiG*	H	Human							
*ldcA*	M	Human*							
*ftsJ*	J	Human							
*nhaA*	C,P	Human*						UTI89 **F11**	(García et al., 2021) **(** Buckles et al., 2015 **)**
*ybeY*	J	Human							
*tolQ*	U	Human*							
*tolABRQ*	U	Human*							
*ompA*	M	Human*							
*mgrB*	S	Human*							
*prc*	O	Human*							
*UTI89_C1262*	J	Human							
*eda*	G	Human							
*rfaDC*	M	Human							
*rfaG*	M	Human			Bladder				
*relA*	K,T	Human							
*recB*	L	Human							
*tusA*	J,O,H	Human							
*atpH*	C	Human*							
*atpF*	C	Human*			Bladder				
***wzxE***	M	Human*			Bladder				
*corA*	P	Human*			Bladder				
*glnA*	K	Human							
*ftsE*	D				Bladder				
*ypdE*	G,E				Bladder				
*himD*	L				Bladder				
*cutA*	P				Bladder				
*phnO*	K				Bladder				
*tamA*	M				Bladder				
*sufA*	O				Bladder*				

^1^Genes could not be validated because of high fitness defects of the single individual mutant strain.

^2^Gene was overexpressed due to a transposon insertion at the promoter region.

^3^Gene also validated in PA45 and UTI89 UPEC strains.

^4^O-antigen gene identified by Sharp et al. (2019) was not directly validated in ST131 EC958; instead, O-antigen density induction in strain MG1655 was used as a comparison (strains L5 and L9).

^5^Cluster of Orthologous Groups (COG) Code: C, Energy production and conversion; D, Cell cycle control, cell division, chromosome partitioning; E, Amino Acid metabolism and transport; F, Nucleotide metabolism and transport; G, Carbohydrate metabolism and transport; H, Coenzyme metabolism and transport; I, Lipid metabolism and transport; J, Translation, ribosomal structure and biogenesis; K, Transcription; L, Replication, recombination and repair; M, Cell wall/membrane/envelop biogenesis; N, Cell motility; O, Post-translational modification, protein turnover, chaperone functions; P, Inorganic ion transport and metabolism; Q, Secondary metabolites biosynthesis, transport and catabolism; R, General function prediction only; S, Function unknown; T, Signal Transduction; U, Intracellular trafficking, secretion and vesicular transport; V, Defense mechanisms; W, extracellular structures.

^6^Genes described in this study were identified using a Multiple round PCR approach.

^7^Functions were assayed in CFT073 by Hirakawa et al. (2019).

*Functions were assayed but could not be confirmed in single specific mutants.

In bold is indicated genes and functions that have been also assayed in single mutants in several studies other than in the TIS study in which the gene was identified. UPEC strain used, and the reference are also highlighted in bold. LPS: Altered LPS patterns compared to WT.

Phan et al. analyzed all the potential genes involved in serum resistance in the UPEC strain EC958 using TraDIS (Phan et al., 2013). In this case, massive parallel sequencing of the generated transposon libraries and further bioinformatics analysis allowed the determination of the essential genome (i.e genes required for growth on LA plates) and the identification of the serum resistome. Most of the genes included in the serum resistome were involved in lipopolysaccharide (LPS) synthesis or encoded membrane proteins. Moreover, 82% of them were further confirmed, by performing site-specific mutagenesis using lambda red (Datsenko and Wanner, 2000) followed by validation studies, to play a role in serum resistance (**Table 1**). Besides LPS, identified as the main resistance mechanism in EC958 in serum, the enterobacterial common antigen and colanic acid biosynthesis were also revealed as relevant for survival in serum. Notably, the authors identified a novel function for two factors as implicated in the regulation of the O-antigen chain length (**Table 1**).

TIS has also been applied to study the course of UPEC systemic infection. The first report used a murine model of bacteremia where a systemic infection with UPEC CFT073 was developed and genes contributing to septicemia were screened for (Subashchandrabose et al., 2013b). Results showed that polysaccharide synthesis and multiple peptide uptake genes, among others, were linked to survival within the murine bladder, spleen, and liver. This study suggested that many of the UPEC fitness mechanisms for survival are the same regardless of the organ. In this study, a total of 9 candidate fitness genes were confirmed to be important in the murine model of systemic infection (**Table 1**).

TIS has also been used to identify genes relevant for colonization of the gut, which often acts as a reservoir from which UPEC can spread and cause disease through fecal-to-urinary tract dissemination (Russell et al., 2017). In this work, it was demonstrated that *glpG*, which encodes a rhomboid protease involved in an unknown cellular process, is vital for colonization and survival in the mouse gut (**Table 1**).

In another study, the authors used an ordered defined transposon mutant library in CFT073 to determine genes important for growth in human urine (Shea et al., 2020). Most of the fitness genes identified were involved in metabolism and DNA damage response. Twelve specific transposon mutants were further tested during growth in human urine (**Table 1**). The defined transposon library, consisting of approximately 9000 mutants, was further used to investigate fitness gene contribution during colonization of the mouse bladder. According to the authors, the relative low number of mutants tested should help overcome the bottleneck effect observed in animal studies, in which Tn-mutants are lost during the infection process as a result of the limitation in simultaneous propagation of different clones in the bladder due to the organ’s anatomy and urination (Walters et al., 2012; Shea et al., 2020). A total of 40 genes were identified as involved in murine bladder colonization combining the TIS analysis and qPCR data, of which only 7 were validated (**Table 1**).

As mentioned above, the bottleneck associated to the traditional UTI murine model represents a limitation when using TIS to analyze fitness-gene contribution during UTI *in vivo*. To circumvent this, in a very recent study, glucose-supplemented water was administered to mice prior to infection. The resulting increased water intake by the animals reduced their urine concentration to levels closer to human urine (García et al., 2021), creating a less hostile milieu which may partially overcome the bottleneck effect and increase the chance of successful infection by the Tn-mutants. Using TraDIS and this modified mouse model of UTI, the authors predicted genes involved in bladder colonization in the UTI89 strain. The role in infection of 10 selected fitness genes, some of them never reported before, was confirmed by targeted mutagenesis and further challenge of mice (**Table 1**). Fitness-gene contribution during growth in human urine was also analyzed and a total of 9 genomic regions were validated as relevant for growth in human urine in UTI89 (García et al., 2021) (**Table 1**). Particularly, the authors revealed *rfaG*, involved in LPS biosynthesis, as required for growth in both niches, and was therefore suggested as potential target for interventions against UTIs caused by UPEC.

#### 3.1.2 TIS Approaches Applied to Study UPEC Motility and Adhesion

Motility and adhesion are key factors for the course of a successful UTI. A previous study revealed known and novel determinants involved in motility of the UPEC strain EC958 using TIS (Kakkanat et al., 2017). In this work, the authors could pinpoint both motility repressors and enhancers by the discovery of insertion polar effects. Moreover, it was found that a widely distributed phage protein in UPEC, EC958_1546, was involved in enhancement of motility in different reference strains, such as UTI89 and CFT073. To date, this is the only published report that has validated TIS findings using multiple UPEC strains (**Table 1**).

To identify factors contributing to the expression of type 1 fimbriae, a construct consisting of a chromosomal transcriptional reporter composed of *lux* under the control of the fimbrial promoter region, *fimS*, was inserted into the UPEC CFT073 genome. Type 1 fimbriae expression modulation was analysed applying TIS in the resulting *fimS* reporter strain and the authors predicted the periplasmic protein, YqhG, to be involved in the expression of such fimbriae (Bessaiah et al., 2019). Moreover, the Δ*yqhG* mutant was confirmed to show reduced expression of type 1 fimbriae and a decreased ability to infect the murine urinary tract (**Table 1**). The mutant was also more motile and showed greater sensitivity to hydrogen peroxide than the WT, suggesting that the gene was also linked to oxidative stress resistance. Thus, *yghG* might represent an interesting novel therapeutic target against UTIs.

#### 3.1.3 Other TIS Studies

Additional TIS-based studies have investigated the molecular mechanisms behind UPEC tolerance towards environmental agents encountered during UTIs. For example, the set of UPEC determinants conferring protection against zinc (Zn) toxicity was studied using TraDIS in the strain EC958 by Stocks et al. (2019). The authors focused on the mechanisms behind innate immune subversion by resistance towards Zn toxicity. Using a saturated transposon mutant library together with validated Zn stress reporter systems, the authors revealed that, in contrast to the reference nonpathogenic *E. coli* K-12 strain MG1655, most cells of EC958 evade the Zn toxicity response within human macrophages, enabling intramacrophage survival. It was also proved that Zn susceptible mutants of EC958 were not compromised for intramacrophage survival while the corresponding K-12 mutants yielded significantly lower bacterial numbers within these cells (**Table 1**). In addition, the study showed that EC958, which is naturally more tolerant to Zn than the K-12 strain, employs both evasion and resistance mechanisms against Zn toxicity, contributing to its systemic dissemination and increased pathogenicity (Stocks et al., 2019).

Other virulence traits have been studied in UPEC using TIS, such as the ability to synthesize the polysaccharide K capsule or the factors involved in macrophage killing and immune evasion. In the first study, authors identified known genes involved in capsule biosynthesis, such as the K1 capsule gene cluster *kpsFEDUCSMT* and *neuDBA* in the urosepsis strain PA45B, as well as two additional regulatory genes (*mprA* and *lrhA*) which were further characterized at the molecular level (Goh et al., 2017) (**Table 1**). In the second one, research into genes that mediate human macrophage cell death revealed that UPEC hemolysin and hemolysin regulators are important contributors to killing of human macrophages (**Table 1**) (Murthy et al., 2018; Nhu et al., 2019). Additionally, several TIS-related studies have aimed to define UPEC molecular mechanisms of resistance to antibiotics frequently used to treat UTIs (Sharp et al., 2019; Phan et al., 2020).

## 4 Identification of UPEC Genes Involved in UTIs Using Transcriptomics

The bacterial transcriptome reflects the organism’s immediate, adaptive response to its environment (Hancock et al., 2008). Over the last decades, transcriptomic-based approaches (including both microarrays and RNA-seq) have been frequently applied to investigate UPEC genes involved in UTIs, revealing strategies that UPEC employ for colonization, growth and survival in the urinary tract and providing insights into the mechanisms that underpin pathogenesis. Compared to other ‘omics’, transcriptomics has been the approach broader applied to this field.

### 4.1 Factors Expressed in UPEC During Human Bacteriuria, Biofilm Formation and Mouse Cystitis

In a report, representing the first assessment of any *E. coli* pathotypes’ transcriptome *in vivo*, the gene expression profiling of the UPEC reference strain CFT073 from urine of *in vivo* infected mice was quantified (Snyder et al., 2004). The *in vivo* transcriptomes were compared to that of UPEC CFT073 grown in rich medium or human filtered urine. Overall, the most highly expressed genes *in vivo* encoded translational functions, suggesting rapid growth of bacteria despite nutrient limitations. Other highly upregulated genes encompassed; those encoding type 1 fimbriae and other adhesion factors, genes involved in iron acquisition, genes responsible for capsular polysaccharide and LPS synthesis as well as genes involved in drug resistance and microcin secretion. However, other fimbrial genes, such as those encoding P and F1C fimbriae, and genes involved in motility and chemotaxis were downregulated during UTI. Transcriptomics of *E. coli* CFT073 in human urine, partially mimicking growth *in vivo*, revealed that genes associated with iron acquisition, capsule, and microcin secretion, were upregulated. As several siderophores like enterobactin or the *sitABCD* system, osmoprotectants such as the betain derivative transporters *proPVWX*, and nitrogen-regulated genes such as *glnA* were upregulated *in vivo*; the authors speculated that the urinary tract might be nitrogen and iron limited, of high osmolarity yielding osmotic stress, and of moderate oxygenation (Snyder et al., 2004).

In order to elucidate determinants that may represent true UPEC virulence factors during UTIs, comparative transcriptomic studies were performed with the asymptomatic bacteriuria (ABU) *E. coli* strain 83972 and UPEC CFT073 (Hancock et al., 2008). The strain 83972 had been confirmed, in a transcriptome-based study, to show mutations in the *foc* locus and therefore is unable to express F1C fimbriae, a primary adhesion factor during UTI (Roos et al., 2006). Transcriptomic analysis of 83972 under seven different environmental conditions including human urine, urine biofilms and human bladders revealed that 96% of the transcripts were present in both 82972 and CFT073 strains (Hancock et al., 2008). Thus, the strains seem to behave very similarly despite yielding very different symptom profiles. However, some of the common genes were only expressed during one or a few of the tested conditions with differences between the three human patients tested, considered as three different environments. Several genes were upregulated in all urine conditions (compared to MOPS) and included, among others, genes involved in iron uptake/transport. Results from this study suggested the harmless *E. coli* strain 83972 to be a deconstructed pathogen and genes expressed exclusively in CFT073 could be considered as virulence factor candidates. Besides, transcriptomic profiling of the biofilms of these strains revealed that functional gene profiles during biofilm formation were very similar (Hancock et al., 2010), although they also demonstrated that biofilm formation is a trait dependent on seemingly minor strain differences. Interestingly, the role in biofilm formation of two upregulated genes, *yhcN* and *ybiJ*, in both strains was confirmed in CFT073 (**Table 2**) and mutants for each gene showed significantly reduced motility compared with the WT strain. In contrast, none of the two 83972-derived mutants, displayed any significant change in biofilm formation compared with the WT. Besides, some iron- and stress -related genes were significantly upregulated in biofilm in both strains. This is an agreement with a previous study, where many genes involved in transcription and stress response were highly expressed in 83972 and in another ABU strain, VR50, during biofilm formation in urine (Hancock and Klemm, 2007). In the latter study, four of the upregulated genes, i.e., *yceP, yqgA, ygiD*, and *aaeX*, were demonstrated to be involved in biofilm formation (**Table 2**). Among others, two potential “true” virulence factors, only upregulated or highly expressed during growth of CFT073 in urine, encoding and iron siderophore receptor and P fimbriae, were identified.

**Table 2:** Validated genes identified by transcriptomics.

Gene	Function (COG)^2^	Validation study						UPEC strain	Reference
		Urine (bacteriuria)	Serum (urosepsis)	Infection of cells (cystitys)	Animal model(bladder, kidney)	Biofilm formation	Other		
*yceP*	S					Human urine		83972 VR50	(Hancock and Klemm, 2007)
*yqgA*	S					Human urine			
*aaeX*	S					Human urine			
*ygiD*	S					Human urine			
*papX*	K				Bladder*, kidney		Increased motility	CFT073	(Simms and Mobley, 2008b)
*yhcN*	S					X	Reduced motility on agar	CFT073	(Hancock et al., 2010)
*ybiJ*	S					X	Reduced motility on agar		
*bssS*	S					X*			
*qseC*	T	X			X		IBC formation, motility	UTI89	(Hadjifrangiskou et al., 2011)
*aceA*	C				X		IBC formation, motility		
*sdhB*	C				X		IBC formation, motility		
*mdh*	C				X		IBC formation, motility		
*pafRP*	G,K	Mouse			Bladder, kidney			CFT073	(Baum et al., 2014)
*pafP*	K	Mouse			Bladder*, kidney	X	Motility		
*kpsC*	M		X					CFT073	(Miajlovic et al., 2014)
*rcsB*	K		X						
*rpoE*	K		X						
*cpxR* ^1^	K		X						
*wcaDE*	S		X						
*yjbE*			X*						
*ydhA*	S		X*						
*rcsA*	K		X*						
*rcsC*	T		X*						
*cus*					X			CFT073	(Subashchandrabose et al., 2014)
*eutR*	K				X				
*fdhF*	C				X				
*kdp*	P				X				
*nik*	P				X				
*phnR*	–				X*				
*tau*	P				X*				
*wcaLM*	M				X*				
*cysB*	K				X*				
*pspA*	K,T						Survival mouse bone marrow-derived macrophages	UTI89	(Mavromatis et al., 2015)
*chuA*	M						IBC formation	UTI89	(Reigstad et al., 2007)
*entF*	Q						IBC formation*		
*hlyA*	Q						IBC formation*		
*iroN*	P						IBC formation*		
*sitA*	P						IBC formation*		
*fyuA*	P				Bladder, kidney, spleen		Growth *in vitro* under iron-replete and iron-limiting	536	(Brumbaugh et al., 2015)
*gspC*	U				X			UTI89	(Conover et al., 2016a)
*srlA*	G				X				
*yeaR*	P			X	X		Type 1 piliation, tellurite and oxidate stress sensitivity		
*lacZ*	G				X				
*galK*	F				X				
*ybdB^#^*	Q								
*mgtS*	–			X	X			CFT073	(Li et al., 2021)

^1^This gene was initially detected in a transcriptomics assay and further validated by Dbeibo et al., 2018, in an animal model (bladder and kidneys).

^2^COG Code: C, Energy production and conversion; F, Nucleotide metabolism and transport; G, Carbohydrate metabolism and transport; K, Transcription; M, Cell wall/membrane/envelop biogenesis; N, Cell motility; P, Inorganic ion transport and metabolism; Q, Secondary metabolites biosynthesis, transport, and catabolism; S, Function unknown; T, Signal Transduction; U, Intracellular trafficking, secretion and vesicular transport.

IBC, Intracellular bacterial communities.

*Genes tested in validation assays whose role could not be confirmed under the tested conditions.

^#^Deletion of the gene was unsuccessful despite several attempts.

In order to investigate how UPEC gene expression in murine models of UTI compares to gene expression during UTI in humans, a study used a UPEC strain CFT073-specific microarray to estimate global gene expression in eight *E. coli* isolates collected and analyzed without further growth *in vitro* from the urine of eight women with UTI (Hagan et al., 2010). The obtained transcriptomic profiles were compared to those of the same *E. coli* isolates cultured in sterile human urine *ex vivo*. Importantly, this study illuminated the metabolic and pathogenic profile of UPEC during human bacteriuria and overall supported the described model of UPEC metabolism during UTI (Anfora et al., 2008; Alteri et al., 2009). Thus, it was speculated that UPEC replicates rapidly during human UTI and replicates faster *in vivo* than *in vitro* with genes encoding ribosomal subunits being the most highly expressed. Other genes indicative of rapid bacterial growth i.e. those encoding transcription and translation machinery, F0F1 ATPase components, fatty acid biosynthesis factors, protein folding and secretion apparatus, outer membrane components and cell division factors were also upregulated. Other genes highly expressed during human UTI included those involved in nucleotide synthesis, LPS assembly, peptidoglycan recycling, enterobacterial common antigen synthesis, iron acquisition, and peptide transport systems. Consistent with transcriptome data from murine UTI, flagellin gene expression was downregulated *in vivo* as compared to *in vitro* culture. Furthermore, as shown in mice, UPEC was speculated to undergo aerobic or microaerobic respiration during human UTI with similar oxygenation suggested *in vivo* (mouse and human) and *in vitro* approaches. Notably, expression of the cytochrome o oxidase genes which are expressed in oxygen-rich conditions, varied among patients and strains. In addition, results revealed that i) UPEC experiences nitrogen limitation also *in vivo* in human, ii) abundant and easily assimilable carbon sources, including peptides, are likely available to UPEC during human cystitis and iii) acetogenic growth might be of relevance. Interestingly, *in vivo* expression of some iron-related genes was isolate-dependent, several other iron-associated genes were not expressed by any strain, and *in vivo* and *in vitro* expression of iron uptake systems did not always correlate. Isolates occasionally expressed a specific system in only one condition. These results suggested, on one hand, that the extent of iron limitation differs among patients, as well as between the human bladder and urine *ex vivo*, and on the other hand, that additional regulation of iron acquisition systems during *E. coli* infection might occur. Notably, although most of the findings were consistent with previous data obtained from the murine UTI model, host-specific differences were detected. Thus, contrary to observations in mice, expression of type 1 fimbriae was undetectable in most of the *E. coli* strains although they were able to express type 1 fimbriae *in vitro* and highly upregulated *fimA* during murine infection. Later studies have suggested that UPEC from human urine samples mainly represents the planktonic sub-population in the bladder and therefore may explain the absence of type-1 fimbriae induction, which are primarily expressed in the sessile population of the bladder epithelial surface (Greene et al., 2015; Stærk et al., 2016). Other fimbriae-related genes were not expressed in any of the *E. coli* strains.

The mentioned studies, although informative, should be interpreted with some care, as the genome of *E. coli* CFT073 was used for comparisons resulting from the hybridization experiments, regardless of the test strain. Thus, gene content and expression may differ between the clinical strains and CFT073 resulting in poor hybridization, leading to misinterpretation of results. Later, an RNA-seq-based study aiming to understand the behavior of UPEC during UTI in women with uncomplicated, naturally occurring UTIs was performed, also, as mentioned above, avoiding *in vitro* growth after collection of the samples (Subashchandrabose et al., 2014). The findings provided insight into the host-specific induction of UPEC fitness genes during human UTI (Subashchandrabose et al., 2014; Mobley, 2016). Thus, comparative transcriptional analysis of UTI samples to the UPEC isolates cultured in human urine and laboratory medium identified novel fitness genes specifically expressed during human UTI. The resulting gene lists encompassed factors involved in ion transport, including copper (Cu) efflux, nickel and potassium import systems, genes linked to uptake and metabolism of ethanolamine, and FHL complex related genes, which were further confirmed as key fitness factors in uropathogenesis using an experimental model of UTI (**Table 2**). In this study, Cu supplementation in drinking water was shown to reduce UPEC colonization of the mouse bladder. Of note, Cu-mediated killing is a key innate immune mechanism used to combat bacterial infection. In UPEC, Cu-resistance includes several mechanisms such as Cus proteins and a Cu-resistance mechanism where the siderophore yersiniabactin, involved in iron acquisition, binds and sequesters Cu (Chaturvedi et al., 2012; Chaturvedi et al., 2014). In another study, RNA-seq analyses of *E. coli* causing cystitis in women demonstrated that iron acquisition systems, including the yersiniabactin system, are highly expressed by bacteria during natural uncomplicated UTI (Brumbaugh et al., 2015). Moreover, blocking yersiniabactin import by deleting the gene encoding its receptor, *fyuA* attenuates UPEC during cystitis, pyelonephritis, and systemic infection in mice (**Table 2**). Thus, the yersiniabactin system represents a promising antibacterial target to treat and prevent UTI (Brumbaugh et al., 2013; Brumbaugh et al., 2015). Overall, upregulation of nickel uptake genes together with the FHL complex genes and the anaerobic Cu-resistance system Cus, demonstrated by Subashchandrabose et al., 2014, suggested that UPEC may adapt to low-oxygen conditions, and anaerobic processes may be involved in promoting fitness during UTI in humans. Importantly, the mentioned study added a new dimension to the model of pathogenesis, highlighting that adaptation to a low-oxygen environment is crucial for successful colonization of the bladder. Interestingly, results showed that urine from healthy volunteers was iron-limited compared with urine from UTI patients, but bioavailability of Fe in urine from patients with UTI might be limited because of sequestration by host factors like hemoglobin, transferrin and albumin (Cassat and Skaar, 2013). In addition, in the patient samples, the phase-variable type 1 fimbriae genes were upregulated in some strains, but not expressed in others (Subashchandrabose et al., 2014).

The aim of the studies mentioned above has been to uncover those virulence factors that are essential for causing UTIs. However, some of them have also revealed that UPEC strains can differ dramatically in expression of specific factors during UTIs, and that the expression of virulence factors by the same strain can differ from patient to patient (Hancock et al., 2008; Hagan et al., 2010; Subashchandrabose et al., 2014). These are relevant observations that should be acknowledged when designing novel therapies. Realizing this, a transcriptomics-based study recently aimed to define a common virulence genotype to UPEC strains causing UTI (Sintsova et al., 2019). This report focused on the core genome using RNA isolated from the urine of 14 patients with uncomplicated UTIs. A core of bacterial genes expressed during different stages of human UTIs was revealed, i.e. a conserved gene expression signature shared by all UPEC strains during UTI was recorded. Notably, in agreement with previous studies, this common transcriptional program underlying UTIs was characterized by upregulation of replication and translational machinery (increase of ribosomal protein expression) allowing UPEC to grow fast within the host (Bielecki et al., 2014; Burnham et al., 2018; Forsyth et al., 2018). The authors pointed out that the increased growth rate is associated with a switch to a more specialized catabolism and scavenging lifestyle in the host. For example, the authors found that during UTI, UPEC downregulates sugar catabolism genes and upregulates amino acid and carbon transporters. Moreover, elevated levels of ribosomal transcripts correlated with decreased level of metabolic gene expression in agreement with the “growth law” (Scott et al., 2010; Basan, 2018), and this reallocation of resources within the core genome might drive the rapid growth rate of UPEC during infection.

As is evident from the above information, UPEC fitness and virulence factors have been assessed through different laboratory approaches over the last decades, including a well-established mouse model of UTI. Nonetheless, how bacterial physiology differs between experimental models and human infections remains unknown. A transcriptomics approach has been recently applied in order to address this relevant question (Frick-Cheng et al., 2020). In this study, transcriptomes of three different UPEC strains in human infection, mouse infection, LB culture, and filter-sterilized urine culture were compared. A strong correlation in UPEC gene expression between the mouse model and human UTI was detected using identical strains. Notably, gene expression of both canonical virulence factors and metabolic machinery were highly similar between the mouse model and human infection. However, the *in vitro* conditions showed remarkable differences, mainly centered in the downregulation of amino acid biosynthesis genes during growth in urine; and upregulation of rRNA synthesis and ribosomal subunit production during both mouse and human infections. The comparison between bacterial gene expression in the mouse model and in human infections showed a significant downregulation of genes involved in anaerobic growth and an upregulation of the D-serine transport system during human UTI. These differences support better UPEC growth in humans and suggest that the human bladder is higher oxygenated and has different nutrient availability than the mouse bladder. Nevertheless, the authors support the continued use of the mouse model for the study of the pathogenesis of human UTI as bacterial gene expression between the mouse model and human UTI strongly correlates when using the same strains. However, the slight differences acknowledged in expression of some genes, which may have a significant impact during infection, are ignored.

### 4.2 Factors Expressed in UPEC During Other Stages of UTI

The transcriptional profile of UPEC has been also analyzed at other UTI stages/niches other than human urine or mouse cystitis, such as IBCs, bladder epithelial cells (BECs), macrophages and serum. The list of upregulated genes within IBCs encompassed genes involved in carbon metabolism, iron acquisition, and transport (Conover et al., 2016a). Overall, the results showed the importance of galactose for UPEC growth within IBCs, as it is thought to be a glucose-limiting environment, forcing UPEC to use an alternative carbon source. In line with this, mutants lacking *lacZ* or *galK* developed significantly smaller IBCs and were attenuated compared to the WT during mice infection (**Table 2**). The study also revealed that bacteria are under oxidative stress within IBCs as the gene *yeaR*, was highly upregulated. In addition to a role in oxidative stress, *yeaR* was also shown to contribute to type 1 fimbriae production, a key factor needed for UPEC invasion of bladder epithelial cells (BECs) (**Table 2**). Another transcriptome-based study indicated that both hemin- and siderophore-associated iron play key roles in IBC development in UPEC (Reigstad et al., 2007). Thus, mutants lacking the gene coding for the hemin receptor *chuA* produced significantly smaller IBCs than the WT UTI89 (**Table 2**). The transcriptome profile of UPEC after invading bladder epithelial cells (BECs) (Li et al., 2021), revealed that among other potential virulence-related genes, *mgtS*, was upregulated and was proved to contribute to UPEC invasion of BECs and colonization of murine bladders (**Table 2**). Expression of *mgtS*, which participates in the accumulation of intracellular magnesium, was confirmed to be activated by the two-component regulatory system PhoPQ, and magnesium limitation was speculated to be a host signal for the activation. The UPEC transcriptional program associated with intramacrophage survival was also analyzed using RNA-seq (Mavromatis et al., 2015). Mouse bone barrow macrophages (BMMs) were challenged for 24 h with two UPEC reference strains UTI89, which survives in BMMs, and 83972, which is killed by BMMs. The transcriptional responses of the UPEC strains diverged markedly from each other, and interestingly, deletion of the UTI89 upregulated gene *pspA* significantly reduced survival within BMMs (**Table 2**). *pspA* belongs to the phage-shock-protein response system, which is involved in the maintenance of the cytoplasmic membrane integrity. Another transcriptomics-based study investigated the transcriptional response of UPEC to human serum (Miajlovic et al., 2014). Serum resistance in CFT073 was mediated by two different protective groups of proteins. One consisted of proteins under the control of the extracytoplasmic stress response regulators CpxR, RpoE, and RcsB. CpxR and RpoE are known to participate in the envelope and periplasmic stress response, while the Rcs two-component system is triggered by peptidoglycan stress. The second protein group consisted of proteins involved in production of exopolysaccharide colanic acid, which in turn is also regulated by RcsB. The contribution of the three envelope stress response regulators *rcsB, rpoE*, and *cpxR* and the region *wcaDE* of the colonic acid operon to serum survival was confirmed experimentally (**Table 2**). The results suggested that the production of colonic acid might be protective while cell wall damage caused by serum components is being repaired. Notably, many of the upregulated genes induced by bactericidal serum were regulated by the Rcs two-component system.

### 4.3 UPEC Genes Expressed in Response to Specific UTI Environmental Factors

Other transcriptomic-based studies have focused on the specific response of UPEC to specific factors encountered during a UTI. Urine contains significant amounts of inorganic ions and urea, which yield osmotic and denaturing stresses on UPEC. Withman et al. investigated the transcriptional adaptive responses of UPEC CFT073 during growth in the presence of 0.3 M NaCl or 0.6 M urea and revealed that the gene expression profiles were drastically different depending on the osmolyte analyzed (Withman et al., 2013). The authors speculated that urea could be a trigger for UPEC to initiate a UTI. Thus, colonization and virulence factors including genes encoding type 1 and F1C fimbriae, genes involved in capsule biosynthesis and several chaperone genes were upregulated in urea supplemented medium but were not stimulated by high salt concentration. On the other hand, adding NaCl to the medium yielded an upregulation of several anaerobic metabolic pathways and overexpression of most of the genes belonging to the osmotic inducible regulon while urea did not induce an osmotic stress response.

Another study applied transcriptomics (RNA-seq) to explore the response of UPEC CFT073 to nitric oxide (NO), which is a toxic gas encountered by UPEC during UTI. The transcriptome of a UPEC triple mutant lacking all known and candidate NO detoxification pathways (Hmp, FlRd and the respiratory nitrite reductase, NrfA) was analyzed in anaerobic cultures grown in the presence and absence of nitrate. Several virulence-associated genes were upregulated when nitrate was present as a source of NO, suggesting that host-derived NO is a potential regulator of UPEC virulence.

### 4.4 Contribution of Specific UPEC Factors to Pathogenesis

Other transcriptomic-related studies revealed how specific genes contribute to fitness/virulence of UPEC. In this context, the role of the sensor kinase QseC in UTI89 pathogenesis was investigated using microarrays (Hadjifrangiskou et al., 2011). Deletion of *qseC* resulted in mis-regulation of nucleotide, amino acid, and carbon metabolism as well as in virulence factor downregulation. Deletion of *qseC* impeded TCA cycle progression, which was shown to be associated with virulence attenuation *in vivo* (**Table 2**). Thus, disruption of metabolic genes affecting the TCA cycle derived in a Δ*qseC*-like phenotype *in vivo* resulting in virulence factor downregulation. Results confirmed the importance of the TCA cycle for uropathogenesis as previously reported (Alteri et al., 2009). Another microarray-based study examined the connection between P fimbriae expression and motility in UPEC CFT073. The authors concluded that synthesis of P fimbriae inversely regulates flagellum synthesis to repress motility *via* PapX (Simms and Mobley, 2008b). Thus, expression of many motility-associated genes was decreased when *papX* was overexpressed and transcription of motility genes was increased in the *papX* mutant compared to the WT. Using microarray analysis, it was demonstrated that expression of *pafR*, a transcription regulator encoded from the *metV* genomic island in UPEC strain CFT073, affects expression of genes related to bacterial virulence, biofilm formation, and metabolism and inhibits biofilm formation and motility (Baum et al., 2014). Besides, disruption of *pafR* was confirmed to impair the ability of UPEC to infect a UTI mouse model (**Table 2**).

## 5 UPEC Fitness and Virulence Factors During UTIs Revealed by Proteomics

The proteome is defined as the entire protein complement of the bacteria present under a given set of circumstances. It is dynamic, reflecting the immediate environment in which it is analyzed. Therefore, proteomics is the large-scale analysis of proteins expressed under specific conditions and at a particular time point. Importantly, posttranslational modifications, which may greatly affect the function of the final gene-product, can only be detected by studying the proteome (Graves and Haystead, 2002; Lo et al., 2017).

### 5.1 Proteomics Applied to the Investigation of the UPEC Outer Membrane Proteome

Several proteomic studies in UPEC have been focused on the CFT073 strain and the investigation of outer membrane proteins (OMPs) since they may play an important key role in bacterial pathogenesis by facilitating direct interactions between bacteria and their surrounding environment. Besides providing insight into how UPEC adapt to specific environmental conditions, these studies have aimed at identifying candidate antigens for vaccine development against UTIs (Cash, 2014).

The first study reporting the use of proteomics in UPEC dates back to 2007, when Alteri and Mobley used two-dimensional gel electrophoresis and tandem mass spectrometry to characterize the OMPs in UPEC CFT073 during growth in human urine (Alteri and Mobley, 2007). Among the 30 OMPs identified, the expression of seven receptors for iron (ChuA, IutA, FhuA, IroN, IreA, Iha, and c2482) was induced in human urine. Thus, results showed that human urine is characterized by being iron-restricted and UPEC copes with this iron limitation by expressing OMPs involved in iron uptake. Furthermore, the study concluded that the identified OMPs might represent surface-exposed vaccine candidates for prevention of UTIs caused by UPEC.

Similarly, Walters and Mobley used two different proteomic approaches to analyze the OM proteome of CFT073 cultured in pooled human urine in an attempt to identify potential vaccine candidates (Walters and Mobley, 2009). The first one was based on the use of a protease to ‘shave’ surface-exposed peptides from the bacterial cell surface followed by mass-spectrometry. The second one was, a more direct approach which implied bacterial labeling with a biotin tag coupled with two-dimensional liquid chromatography-tandem mass-spectrometry. The latter method showed the best results and allowed rapid identification of the OMPs. Twenty-five OMPs were predicted using this approach, eight of which (FhuA, FepA, IroN, Hma, IutA, ChuA, TonB and C5174) were related to iron transport systems or putative iron-regulated virulence proteins, supporting previous findings on the relevance of iron uptake during growth in urine (Alteri and Mobley, 2007; Walters and Mobley, 2009).

Wurpel et al. applied a high-throughput approach based on tandem mass-spectrometry (LC-MS/MS) of EDTA heat-induced outer membrane vesicles to investigate the OM proteome of five UPEC strains (CFT073, UTI89, UMN026, 536 and F11) during growth in pooled human urine (Wurpel et al., 2016). This approach had been previously performed by the authors to analyze the surface-proteome based on larger UPEC strain collections during growth in M9 media (Wurpel et al., 2015). Overall, several OMPs were detected in all five proteomes. Eleven different iron uptake systems were identified with only three of them shared by the five UPEC strains, and four distinct fimbrial types, including type 1, P and F1C/S were also found. Interestingly, an uncharacterized fimbrial type, UCA-like (UCL) fimbriae, phylogenetically related to the Uca fimbriae of *Proteus mirabilis*, was identified, and further analyses demonstrated its role in biofilm formation and adhesion to human uroepithelial cells (**Table 3**) (Wurpel et al., 2016).

**Table 3:** Validated genes identified by proteomics.

Gene	Function (COG)^2^	Validation study						UPEC strain	Reference
		Urine (bacteriuria)	Serum (urosepsis)	Infection of cells (cystitys)	Animal model(bladder, kidney)	Biofilm formation	Other		
*dppA*	E				X			CFT073	(Alteri et al., 2009)
*oppA*	E				X				
*tpiA*	F				X				
*speB*	E				X				
*sdhB*	C				X				
*pckA*	F				X				
*talA*	F				X*				
*xylA*	G				X*				
*serA*	E				X*				
*uxuA*	G				X*				
*nanA*	H				X*				
*argG*	F				X*				
*araF*	G				X*				
*edd*	E,G				X*				
*gnd*	F				X*				
*pgi*	F				X*				
*uclABCD*	–			X		X	Fimbrial production cell surface	536	(Wurpel et al., 2016)
*chuA^1^*	M	X*						CFT073	(Torres et al., 2001; Alteri and Mobley, 2007; Walters and Mobley, 2009; Wurpel et al., 2016)
*cycA^1^*	E	X*						CFT073 **UTI89**	(Walters and Mobley, 2009; Hibbing et al., 2020)
*carB^1^*	F	X						CFT073	(Vejborg et al., 2012; Wurpel et al., 2016; Shea et al., 2020)
*fdnG^1^*	C	X*						CFT073 **UTI89**	(Walters and Mobley, 2009; Hibbing et al., 2020)
*fhuA^1^*	P	X*						CFT073 **UTI89**	(Alteri and Mobley, 2007; Walters and Mobley, 2009; Wurpel et al., 2016) (Hibbing et al., 2020)
*lpp^1^*	M	X						CFT073	(Walters and Mobley, 2009; Diao et al., 2017)
*ompC^1^*	M	X*						CFT073 **UTI89**	(Alteri and Mobley, 2007; Walters and Mobley, 2009; Wurpel et al., 2016) (Hibbing et al., 2020)
*ompF^1^*	M	X*						CFT073 **UTI89**	(Alteri and Mobley, 2007; Walters and Mobley, 2009; Wurpel et al., 2016) (Hibbing et al., 2020)
*pal^1^*	M	X*						CFT073	(Walters and Mobley, 2009; Diao et al., 2017)
*purA^1^*	F	X						CFT073 **Ec83972**	(Vejborg et al., 2012; Wurpel et al., 2016)
*tonB^1^*	U	X*						CFT073	(Torres et al., 2001; Walters and Mobley, 2009)
*yfgM^1^*	S	X						CFT073	(Vejborg et al., 2012; Wurpel et al., 2016)
*xseA^1^*	L	X						CFT073 **UTI89**	(Walters and Mobley, 2009; Hibbing et al., 2020)

^1^Genes detected by proteomics but validated in other studies using similar conditions. In bold is indicated the UPEC strain used for the validation assays when it differs from the UPEC strain employed in the proteomics analysis.

^2^COG Code: C, Energy production and conversion; E, Amino Acid metabolism and transport; F, Nucleotide metabolism and transport; G, Carbohydrate metabolism and transport; H, Coenzyme metabolism and transport; L, Replication, recombination and repair; M, Cell wall/membrane/envelop biogenesis; P, Inorganic ion transport and metabolism; S, Function unknown; U, Intracellular trafficking, secretion and vesicular transport.

*Genes tested in validation assays whose role could not be confirmed under the tested conditions.

### 5.2 Proteomics Applied to Research Into UPEC Metabolism

The knowledge of bacterial metabolism during infection is crucial to better understand the infectious disease mechanism. Thus, Alteri et al. (2009) applied comparative proteomics to investigate the expression of UPEC CFT073 cytoplasmic proteins during growth in human urine in order to study the role of metabolism in bacterial pathogenesis (Alteri et al., 2009). Results showed that proteins required for the import of peptides, the transport and catabolism of sialic acid, gluconate, and the pentose sugars (xylose and arabinose), as well as proteins related to the non-oxidative pentose phosphate pathway, gluconeogenesis and amino acid metabolism (arginine and serine), were significantly induced during human bacteriuria compared to growth in LB iron-limited medium. Further, genes encoding TpiA (gluconeogenesis), SpeB (arginine metabolism and putrescine biosynthesis), SdhB (tricarboxylic acid cycle -TCA-), DppA and OppA (peptide import) proteins were confirmed to be important for fitness in the UTI murine model (**Table 3**). Thus, the results suggested that import of peptides, gluconeogenesis and the TCA are of relevance during growth in urine, and peptides and amino acids represent the primary carbon sources for UPEC during infection of the urinary tract.

Sabarly et al. applied quantitative proteomics to monitor and compare enzymatic activities associated to central metabolism in five *E. coli* strains (three commensal and two pathogenic, including CFT073) during growth in four culture media; minimal medium with either glucose or gluconate, LB and urine (Sabarly et al., 2016). Importantly, this study revealed that nutrients available in each environment drive the metabolic response by arranging changes in abundances of proteins and enzymatic activities belonging to the same pathway category. Thus, regardless of the strain, each culture medium induces a specific oxidative response, while differences detected between strains involve specific proteins and enzymes within pathway categories in each environment. The study suggested a multiplicity of evolutionary strategies between the strains; for ex. UPEC CFT073 showed a deregulation of iron demand and an increased oxidative stress response.

In a recent study, a proteomic approach to predict the metabolic adaptation of UPEC UTI89 at two different stages of a UTI; growth in human urine and invasion of human epithelial bladder cells as well as during growth in lab medium (MOPS) was performed (Mortensen, 2021). Significantly overexpressed proteins in urine compared to growth in MOPS included those involved in iron uptake and amino acid biosynthesis. Similarly, during invasion of bladder cells, proteins associated to iron acquisition and arginine metabolism were overexpressed together with proteins related to sulphur compound turnover. Results suggested that UPEC encounters a richer environment within bladder cells than in urine. Among others, proteins expressed in the bladder intracellular environment compared with urine were involved in purine metabolism. This study predicted how UPEC metabolism differs between the different growth conditions and several novel proteins contributing to UPEC growth during UTIs were identified. However, lack of correlation between protein expression and published gene essentiality in UTI indicates that high level of protein expression does not necessarily mean that such proteins are essential for growth under the tested conditions.

### 5.3 Other Proteomic Studies

A previous study compared the cellular proteomes of *E. coli* isolates collected from cases of UTIs and random fecal samples (Smith et al., 2011) in an attempt to locate variable proteins expressed by the bacteria. The analysis showed that protein profiles from *E. coli* recovered from the same patient were similar regardless of the source, indicating relatively homogenous intestinal and urinary *E. coli* populations, but profiles differed from patient to patient. Selected protein spots were identified by peptide fragment fingerprinting to understand the basis of the bacterial diversity in spot abundance and mobility of proteins. Overall, the results suggested that it may not be a homogenous *E. coli* type that causes UTIs, and in addition, some key proteomic features among clinical *E. coli* isolates that promote infection *via* the urinary tract were defined.

Using matrix-assisted laser desorption/ionization time-of-flight imaging mass spectrometry (MALDI TOF IMS), the spatial proteome of surface-associated biofilms formed by UPEC UTI89 was analyzed (Floyd et al., 2015). Results revealed that two adhesion factors were crucial for biofilm formation and virulence. Type 1 fimbriae (Fim) and curli fibers were expressed at different locations within the biofilms; curli fibers were detected at the air-liquid interface, while type 1 pili localized to the air-exposed region. The study also proved that lack of oxygen turn off the production of type 1 pili, essential for adherence to host bladder cells and catheters (Floyd et al., 2015). Besides providing knowledge on the spatial regulation of proteins within bacterial biofilms, the study identified pathways that may represent targets to inhibit bacterial adherence.

In another study, the extracellular vesicle (EV) proteomes of two *E. coli* strains; UPEC 536 and the probiotic Nissle 1917 were analyzed (Hong et al., 2019). EVs are released into the extracellular milieu (they are found in all bodily fluids, including urine) and play important roles in bacterial survival and pathogenesis (Barreiro and Holthofer, 2017). In this work, the proteome of crude EVs and the proteome from bacterial cultures grown in iron-restricted and iron-supplemented conditions were compared. Overall, it was shown that OMPs were highly abundant in the EV proteomes of the two strains, with certain strain specific differences. It was also concluded that EV proteomes included proteins involved in pathogenicity, and that several EV proteins differed in their abundance depending on the iron availability in the media.

## 6 UPEC Virulence and Fitness Factors Involved in UTIs Identified Through Metabolomics Approaches

Metabolites are small molecules that are transformed during different biological metabolic routes. The comprehensive analysis of these molecules is known as metabolomics, a powerful technique that provides functional information of cellular processes, thereby linking cellular pathways with their biological mechanisms (Patti et al., 2012).

### 6.1 Importance of Iron Acquisition/Siderophores During UTI Revealed by Metabolomics Assays

Most of the metabolomics studies applied in the context of UPEC aim to identify factors and/or mechanisms that might distinguish UPEC from non-pathogenic strains. Some studies have been focused on siderophore biosynthesis since this is considered important for the capability of UPEC to cause a UTI (Henderson et al., 2009; Yan et al., 2015; Su et al., 2016a; Su et al., 2016b). The high-pathogenicity island (HPI) carries the virulence genes responsible for the synthesis, transportation, and regulation of yersiniabactin, a siderophore commonly present in pathogenic *E. coli* strains and usually absent in non-pathogenic *E. coli* (Yan et al., 2015). Yan et al. applied a targeted metabolomics approach combined with a genetic approach to elucidate how HPI regulates central carbon metabolism in UPEC. Surprisingly, the results revealed that the genes present in the HPI allow for flexible adaptation of UPEC to several growth environments, and that the UPEC strain UTI89 displays remarkable metabolic homeostasis compared to a non-UPEC strain (Yan et al., 2015). Another study based on a quantitative metabolomics approach reported that UPEC isolates preferentially expressed yersiniabactin and salmochelin siderophores in contrast to non-UPEC rectal strains (Henderson et al., 2009). Su et al. aimed to investigate how the metabolomes of UPEC and non-UPEC strains could be used to differentiate the two groups of bacteria, and to establish the differential regulatory roles of siderophore biosynthesis on the primary metabolism (Su et al., 2016b). They observed remarkable differences between the identified metabolomes, which therefore allowed to clearly differentiate UPEC from non-UPEC isolates. Data from metabolome assays of mutants for siderophores revealed that the simultaneous lack of all four siderophores; aerobactin, salmochelin, yersiniabactin, and enterobactin, had the highest impact on the regulatory role of the metabolomes of UPEC and non-pathogenic *E. coli* strains. The modulatory role of siderophores mainly affected amino acid metabolism, oxidative phosphorylation in the carbon fixation pathway, and purine and pyrimidine metabolism (Su et al., 2016a). Another study confirmed that the interactive metabolome between UPEC and human urine strongly differed from that of non-UPEC strains with human urine and further demonstrated that biosynthesis of siderophores modulated the interactive metabolome. As such, results supported that siderophore production represents a key factor of UPEC virulence and enables to distinguish UPEC from non-UPEC (Su et al., 2016b). Recently, a metabolomic-based assay identified a novel siderophore encoded in HPI in UPEC isolates; the metabolite HPTzTn-COOH. However, its role in pathogenesis still remains unknown (Xu et al., 2019).

### 6.2 Other Metabolomics Studies

*E. coli* ABU strains yielding asymptomatic bacteriuria, due to their ability to colonize the urinary tract, are difficult to distinguish from those causing UTIs. Thus, a recent study applied metabolomics to identify different metabolic features between ABU and UPEC isolates and revealed purine metabolism as one distinct signature. Thus, UPEC strains can satisfy their purine needs by only salvaging adenosine *via* conversion to xanthosine, and eventually guanosine instead of relying on *de novo* synthesis pathways (Eberly et al., 2020).

Metabolomics approaches have also been applied to identify metabolites and metabolic pathways underlying biofilm formation in UPEC. The metabolism of biofilm and planktonic populations were compared, and results revealed different metabolic traits depending on the condition. Thus, several differentially produced metabolites were measured, thereby identifying three metabolic pathways related to glycerolipid, amino acid biosynthesis and carbohydrate metabolism(s) as being enhanced during biofilm formation (Lu et al., 2019).

## 7 Other ‘Omics’ Studies Applied to Identify UPEC Factors Contributing to UTIs

Besides the mentioned ‘omics’ approaches, there are other less commonly used ‘omics’-related techniques which have also been applied to identify UPEC factors contributing to UTIs.

The application of an immunoproteomics approach to vaccine development against UTIs has been described, aiming at detecting antigens that stimulate a humoral response during experimental UTI with UPEC CFT073 (Hagan and Mobley, 2007). Outer membranes were isolated from the strain cultured under UTI-related conditions and further probed using pooled antisera from mice chronically infected with UPEC CFT073. Among the outer membrane antigens reacting with the antisera, a novel iron receptor (c2482), and other iron-associated proteins: ChuA, IroN, IreA, Iha and IutA, were identified. Some of them were confirmed to be highly conserved among UPEC strains and could represent potential candidates for a vaccine to prevent UTIs.

Another study applied DFI-Seq that combines differential fluorescence induction (DFI) with NGS to identify differentially expressed genes in UPEC during growth under UTI-associated conditions (Madelung et al., 2017). This technique consists in using a *gfp*-based promoter trap mutant library to infect a tissue culture model following by isolation and enrichment of GFP-activated individuals using fluorescence-activated cell sorting prior to high-throughput sequencing. Results showed that genes involved in amino acid biosynthesis were upregulated during growth in human urine. Furthermore, mutants lacking genes involved in arginine biosynthesis were outcompeted by the WT during growth in human urine and showed reduced ability to infect human bladder cells. During the invasion of bladder cells, 60% of the induced genes encoded hypothetical proteins, and a mutant lacking the gene UTI89_C5139, showed increased adhesion and invasion capabilities (Madelung et al., 2017).

Chromatin immunoprecipitation followed by sequencing (ChIP–seq) allows for genome-wide profiling of DNA-binding proteins, histone modifications, or nucleosomes. Thus, ChlP-seq enables the identification of binding sites for transcription factors, core transcriptional machinery and other DNA-binding proteins which is crucial for interpreting the gene regulatory networks that trigger biological processes (Park, 2009). As previously mentioned, results from transcriptomics in UPEC in response to NO revealed that the list of the most highly upregulated genes included some known to be regulated by NsrR (Mehta et al., 2015), a transcriptional factor in *E. coli* involved in the response to NO. In the same study, by using ChlP-seq, the authors assessed the NsrR regulon in CFT073 and identified several NsrR binding sites in promoter regions in the CFT073 genome, approximately 60% of which were not previously identified in *E. coli* K-12. Therefore, the authors suggested that NsrR may regulate some CFT073 genes that do not have homologues in *E. coli* K-12.

In another study, a metallomics-based study using a liquid chromatography mass spectrometric approach was carried out to investigate the metal selectivity by the siderophore yersiniabactin and its membrane importer FyuA (Koh et al., 2015). Besides binding copper, iron and gallium ions, the siderophore was found to form stable ion complexes with multiple non-ferric metal ions including nickel, cobalt, and chromium. It was shown that these complexes could be imported by the TonB-dependent transporter FyuA into UPEC and among them, only copper(II)-yersiniabactin did not competitively inhibit iron(III)-yersiniabactin import, which suggests that the yersiniabactin system prioritizes iron uptake in copper-rich environments.

## 8 Concluding Remarks

In the last decades, we have witnessed an explosion of ‘omics’-based studies in UPEC. Among the different techniques, NGS and mass spectrometry combined with advanced bioinformatics tools have been the key for obtaining large amounts of data. These technologies have increased our understanding of UPEC biology and pathogenesis in the context of UTIs (Lo et al., 2017).

The goal of these 'omics'-based studies has been mostly dual; to understand the infection process and to identify novel targets for prevention or treatment strategies against the disease. Many of these studies have revealed hundreds of genes and/or proteins that may be of importance for the ability of UPEC to cause UTIs. It is worth to highlight that while transcriptomics and proteomics related works yield valuable information on the lifestyle of UPEC during infection, they do not inform on essentiality. There are numerous examples of redundancy for the most highly up-regulated genes and proteins, such as the systems involved in iron scavenging from the host (Alteri and Mobley, 2007; Hancock and Klemm, 2007; Reigstad et al., 2007; Henderson et al., 2009; Hagan et al., 2010; Chaturvedi et al., 2012; Chaturvedi et al., 2014; Yan et al., 2015; Conover et al., 2016a; Sabarly et al., 2016; Su et al., 2016a; Wurpel et al., 2016). Therefore, for the identification of targets to prevent growth or infection, results from TIS-related studies provide the most immediately useful information.

Interestingly, some of these ‘omics’-based studies have shown that the set of factors contributing to UTIs varies considerably depending on the host-environment. This is relevant in relation to interpretation of results obtained in different animals, cell and *ex vivo* models of UTI, as well as between human patients (Hancock et al., 2008; Hagan et al., 2010; Smith et al., 2011; Subashchandrabose et al., 2014; Wurpel et al., 2015; Sabarly et al., 2016; García et al., 2021). Results suggest that UPEC evolution has equipped the bacterium with the required flexibility to survive and grow despite the variation in the environments encountered during UTI in different hosts. Urine is particularly complex, and its composition varies considerably between patients, and over time in the same patient, depending on diet, fluid intake and inflammatory responses (Mann et al., 2017; García et al., 2021). This fact places heavy demands on UPEC, requiring a flexible and adaptive metabolism and an ability to quickly change survival strategy to persist in the urinary tract. The detailed sensing of the environment by UPEC during UTIs and the adaptive regulation of its gene expression have, so far, only been characterized superficially, but the increasingly advanced ‘omics’ approaches and bioinformatics techniques are powerful tools that likely will assist scientists in shedding light on this in the near future. Similarly, strain-differences have been detected during growth under relevant UTI conditions, confirming that UPEC is a genetically diverse group of bacteria (Hancock et al., 2008; Hagan et al., 2010; Subashchandrabose et al., 2014; Barreiro and Holthofer, 2017; Kakkanat et al., 2017; García et al., 2021). It is unclear to what extend the differences in study design, techniques and statistical analysis of the large data sets have added to this apparent variation between hosts and isolates. Care should be taken when drawing conclusions across models and ‘omics’ approaches, until this is well understood.

Despite the discrepancies detected at the host and strain level, common factors to all studies regardless of the isolate, host or ‘omics’ approach have been identified. Thus, LPS biosynthesis, siderophore production, expression of adhesion factors and certain metabolic genes have come out as important during UTIs (**Table 4**). This has been deduced from both *in vitro* and *in vivo* studies involving different ‘omics’ techniques. Components of the identified systems are the most likely targets for novel prevention/treatment strategies against UTIs. Before considering this, however, it is important that validation assays have been performed with mutants in the selected systems. This review has put emphasis on factors which have been identified as relevant for virulence and fitness in UPEC *via* ‘omics’ approaches, and especially those that have been validated as relevant at different stages of a UTI. This should help future studies in the selection of determinants which may be further investigated as potential target candidates for therapeutics and prophylactics towards UTIs caused by UPEC.

**Table 4:** Validated genes identified by more than one -omics approach during any of the UTI stages.

Gene	Function (COG)^1^	‘Omics’ approach^2^	Reference
*aceE*	F	TN, TR, P	(Hagan et al., 2010; Wurpel et al., 2016; Shea et al., 2020)
*ahpC*	O	TR, P	(Hagan et al., 2010; Wurpel et al., 2016)
*argG*	E	TN, P	(Shea et al., 2020; Alteri et al., 2009)
*atpA*	F	TN, P	(Alteri et al., 2009; García et al., 2021)
*atpD*	F	TN, P	(Walters and Mobley, 2009; García et al., 2021)
*atpE*	C	TN, TR, P	(Walters and Mobley, 2009; Hagan et al., 2010; García et al., 2021)
*atpF*	C	TN, TR	(Hagan et al., 2010; García et al., 2021)
*carB*	F	TN, P	(Wurpel et al., 2016; Shea et al., 2020)
*chuA*	M	TR, P	(Alteri and Mobley, 2007; Walters and Mobley, 2009; Hagan et al., 2010; Wurpel et al., 2016; Conover et al., 2016a)
*cydA*	C	TR, P	(Walters and Mobley, 2009; Hagan et al., 2010)
*damX*	D	TN, P	(Walters and Mobley, 2009; Phan et al., 2020)
*deaD*	F	TR, P	(Snyder et al., 2004; Walters and Mobley, 2009; Hagan et al., 2010)
*dnaK*	O	TR, TN	(Hancock and Klemm, 2007; Nhu et al., 2019)
*efp*	J	TN, TR	(Hagan et al., 2010; Shea et al., 2020; García et al., 2021)
*eno*	F	TR, P	(Hagan et al., 2010; Wurpel et al., 2016)
*fadL*	M	TN, P	(Alteri and Mobley, 2007; Walters and Mobley, 2009; Russell et al., 2017)
*fliC*	N	TN, P	(Alteri and Mobley, 2007; Shea et al., 2020)
*fusA*	J	TN, TR, P	(Hagan et al., 2010; Wurpel et al., 2016; Shea et al., 2020)
*gapA*	F	TR, P	(Hagan et al., 2010; Wurpel et al., 2016)
*gidA*	D	TN, TR	(Snyder et al., 2004; Hagan et al., 2010; Shea et al., 2020)
*glnA*	F	TN, TR	(Hagan et al., 2010; García et al., 2021)
*guaA*	F	TN, TR	(Hagan et al., 2010; García et al., 2021)
*guaB*	F	TN, TR	(Hagan et al., 2010; García et al., 2021)
*iroB*	C, G	TR, P	(Alteri and Mobley, 2007; Conover et al., 2016a; Snyder et al., 2004)
*iroN*	P	TR, P	(Snyder et al., 2004; Alteri and Mobley, 2007; Walters and Mobley, 2009; Wurpel et al., 2016)
*leuB*	C, E	TN, P	(Wurpel et al., 2016; Shea et al., 2020)
*lpdA*	C	TR, P	(Hagan et al., 2010; Wurpel et al., 2016)
*lpp*	M	TN, P	(Walters and Mobley, 2009; Phan et al., 2013)
*metF*	E	TN, TR	(Shea et al., 2020; Li et al., 2021)
*metR*	K	TN, TR	(Shea et al., 2020; Li et al., 2021)
*nhaA*	P	TN, TR	(Mavromatis et al., 2015; García et al., 2021; Li et al., 2021)
*nmpC*		TN, P	(Wurpel et al., 2016; Alteri et al., 2009; Bessaiah et al., 2019)
*ompA*	M	TN, TR, P	(Alteri and Mobley, 2007; Walters and Mobley, 2009; Wurpel et al., 2016; García et al., 2021)
*oppA*	E	TN, P	(Alteri et al., 2009; Walters and Mobley, 2009)
*pdhR*	K	TN, TR	(Alteri et al., 2009; Walters and Mobley, 2009; Subashchandrabose et al., 2013b)
*pstC*	P	TR, P	(Walters and Mobley, 2009; Li et al., 2021)
*purA*	F	TN, P	(Wurpel et al., 2016; Shea et al., 2020)
*rfe*	M	TN, TR	(Hadjifrangiskou et al., 2012; Shea et al., 2020)
*rplN*	J	TR, P	(Walters and Mobley, 2009; Subashchandrabose et al., 2013b)
*rplQ*	J	TR, P	(Walters and Mobley, 2009; Hagan et al., 2010)
*rpoA*	K	TR, P	(Hagan et al., 2010; Alteri et al., 2009)
*rpoE*	K	TN, TR	(Miajlovic et al., 2014; Mavromatis et al., 2015; García et al., 2021)
*rpsI*	J	TN, P	(Walters and Mobley, 2009; García et al., 2021)
*slp*	M	TN, P	(Walters and Mobley, 2009; Bessaiah et al., 2019)
*sspA*	K	TN, P	(Walters and Mobley, 2009; Phan et al., 2013)
*surA*	O	TN, P	(Alteri et al., 2009; García et al., 2021)
*tolC*	M	TN, P	(Alteri and Mobley, 2007; Walters and Mobley, 2009; Nhu et al., 2019; Shea et al., 2020)
*tpiA*	F	TR, P	(Hagan et al., 2010; Alteri et al., 2009)
*tufB*	J	TR, P	(Walters and Mobley, 2009; Hagan et al., 2010)
*uxuA*	G	TR, P	(Wurpel et al., 2016; Alteri et al., 2009; Snyder et al., 2004)
*uxuB*	G	TR, P	(Snyder et al., 2004; Wurpel et al., 2016)
*wcaF*	S	TN, TR	(Phan et al., 2013; Miajlovic et al., 2014)
*yceD*	S	TN, TR	(Hancock and Klemm, 2007; Hagan et al., 2010; García et al., 2021)
*yciB*	D	TN, TR	(Stocks et al., 2019; Frick-Cheng et al., 2020)
*yeaY*	M	TR, P	(Walters and Mobley, 2009; Mavromatis et al., 2015)
*yfaZ*	S	TR, P	(Walters and Mobley, 2009; Frick-Cheng et al., 2020)
*yfgM*	S	TN, P	(Alteri and Mobley, 2007; Shea et al., 2020)
*yhbC*		TN, TR	(Hagan et al., 2010; García et al., 2021)
*yncE*	S	TR, P	(Snyder et al., 2004; Wurpel et al., 2016)

^1^COG Code: C, Energy production and conversion; D, Cell cycle control, cell division, chromosome partitioning; E, Amino Acid metabolism and transport; F, Nucleotide metabolism and transport; G, Carbohydrate metabolism and transport; J, Translation, ribosomal structure and biogenesis; K, Transcription; M, Cell wall/membrane/envelop biogenesis; N, Cell motility; O, Post-translational modification, protein turnover, chaperone functions; P, Inorganic ion transport and metabolism; S, Function unknown.

^2^P, proteomics; TN, TraDIS; TR, transcriptomics.

One of the main challenges to human health is the increasing prevalence of AMR – also among UPEC strains. This fact calls for alternatives to antimicrobials in treatment and prevention of UTIs, and for the development of novel antimicrobials. The path from knowledge on host-pathogen interaction, which has been greatly increased because of the many excellent ‘omics’-based studies, to novel treatment/prophylactic strategies (i.e. novel antimicrobials or generation of potential vaccines) is not straightforward. The many putative targets that have been identified in genome- and proteome -wide studies must be further assessed and tested in dedicated infection models. The first step towards suitable vaccines against UTIs was taken some years back (Mobley and Alteri, 2015; Habibi et al., 2017), but to the knowledge of the authors, such vaccines have so far not been assessed in clinical trials. With regard to novel antimicrobials, the pharma industry generally prefers drug-based rather than target-based screens to search for lead molecules (Lewis, 2020), and due to this, the many putative therapeutic targets may be of little relevance to new antimicrobial discovery.

As mentioned, ‘omics’ works have concluded that the contribution of UPEC virulence and fitness factors to UTIs may vary depending on the strain and host tested. Thus, one main challenge may be to carefully investigate the large data sets and identify common factors among UPEC strains and across hosts. In this respect, the work published by Beielcki et al., who attempted to establish a common *in vivo* virulence gene expression pattern to UPEC strains (Bielecki et al., 2014), may be considered as a model approach, which can help identifying the factors which are shared across different UPEC strains and required during UTI in different patients.

Whole genome sequencing of UPEC strains has led to hundreds of available genomes from different strains around the world; currently, Enterobase contains 1,055 entries when the “Simple Disease” field is UTI. This fact facilitates the screening of factors that are shared across UPEC strains, but it does not solve the problem of the high number of genes encoding hypothetical proteins. Such genes are commonly revealed during analysis of data obtained from ‘omics’ reports, whether transposon, expression or protein based. ‘Omics’ studies have changed the focus from single gene characterization to overview of general pathways and systems involved in UTIs. While this is indeed crucial for the comprehension of the “system” as a whole, it is also of importance to characterize genes with unknown function to reach a full understanding of the different genetic circuitries.

‘Omics’ studies with focus on characterization of bacterial gene and protein expression during UTI are technically demanding. Host RNA and proteins are far in excess compared to bacterial RNA and proteins. Techniques to overcome this problem are still in their infancy.

Our current view of UPEC lifestyle in relation to UTIs has mainly been informed from ‘omics’ studies (global transcription and global proteomes) on UPEC obtained from the urine of infected patients. This may not represent the full picture of the adaptation that UPEC undergoes in relation to UTIs. Furthermore, our current view on UPEC pathogenicity may be partly influenced by variation in the methods used by different research groups, including the use of different UTI *in vitro* and *in vivo* models. Thus, on the methodology side, emphasis should be on improving our ability to study UPEC in the real infection situation, and on improving the efforts in the society to validate not only own observations from ‘omics’ studies, but also to validate main conclusions from other key studies, whenever novel methods to study UPEC *in vivo*, are developed.

## Author Contributions

VG and ST-P retrieved the articles, tabulated findings, and drafted the review. KS, TA, and JM-J drafted and proofread the manuscript. JO provided intellectual inputs and proofread the manuscript. AH-F conceptualized and conceived the study, provided intellectual inputs, analyzed the data, guided inclusion of specific information, retrieved the articles, drafted, proofread the manuscript and approved the final version of the review. All authors contributed to the article and approved the submitted version.

## Funding

This work has been supported by the Danish Research Council for Independent research, grant no. DFF-4184-00050. VG acknowledges the Consellería de Cultura, Educación e Ordenación Universitaria, Xunta de Galicia for her post-doctoral grant (Grant Number ED481B-2018/018).

## Conflict of Interest

The authors declare that the research was conducted in the absence of any commercial or financial relationships that could be construed as a potential conflict of interest.

## Publisher’s Note

All claims expressed in this article are solely those of the authors and do not necessarily represent those of their affiliated organizations, or those of the publisher, the editors and the reviewers. Any product that may be evaluated in this article, or claim that may be made by its manufacturer, is not guaranteed or endorsed by the publisher.
